# Noble Gas Binding Ability of an Au(I) Cation Stabilized by a Frustrated Lewis Pair: A DFT Study

**DOI:** 10.3389/fchem.2020.00616

**Published:** 2020-07-21

**Authors:** Manas Ghara, Pratim Kumar Chattaraj

**Affiliations:** ^1^Department of Chemistry and Center for Theoretical Studies, Indian Institute of Technology, Kharagpur, India; ^2^Department of Chemistry, Indian Institute of Technology Bombay, Mumbai, India

**Keywords:** frustrated lewis pair, noble gas binding, noble gas-noble metal bond, bond dissociation energy, energy decomposition analysis

## Abstract

The noble gas (Ng) binding ability of a monocationic [(FLP)Au]^+^ species has been investigated by a computational study. Here, the monocationic [(FLP)Au]^+^ species is formed by coordination of Au(I) cation with the phosphorous (Lewis base) and the boron (Lewis acid) centers of a frustrated Lewis pair (FLP). The bonds involving Au and P, and Au and B atoms in [(FLP)Au]^+^ are partially covalent in nature as revealed by Wiberg bond index (WBI) values, electron density analysis and energy decomposition analysis (EDA). The zero point energy corrected bond dissociation energy (D_0_), enthalpy and free energy changes are computed for the dissociation of Au-Ng bonds to assess the Ng binding ability of [(FLP)Au]^+^ species. The D_0_ ranges from 6.0 to 13.3 kcal/mol, which increases from Ar to Rn. Moreover, the dissociation of Au-Ng bonds is endothermic as well as endergonic for Ng = Kr-Rn, whereas the same for Ng = Ar is endothermic but exergonic at room temperature. The partial covalent character of the bonds between Au and Ng atoms is demonstrated by their WBI values and electron density analysis. The Ng atoms get slight positive charges of 0.11–0.23 |*e*|, which indicates some amount of charge transfer takes place from it. EDA demonstrates that electrostatic and orbital interactions have equal contributions to stabilize the Ng-Au bonds in the [(FLP)AuNg]^+^ complex.

## Introduction

The noble gas (Ng) elements (He, Ne, Ar, Kr, Xe, and Rn) were have been supposed to be non-reactive in forming chemical bonds with other elements in the periodic table. Their inertness arises because the s and p orbitals are totally occupied. However, Pauling (1933), predicted the possibility of bond formation by the heavier Ng elements. Since the core electrons will exhibit larger screening effect for the valence electrons on moving down the group, the valence electrons would be in a relatively loosely bound state and hence they can be easily ionized. However, it took long time to convert it into reality when xenon hexafluoro platinate [Xe^+^(PtF_6_)^−^] was synthesized by Bartlett (1962). This discovery opened a new chapter in the Ng chemistry refuting the prejudice regarding the chemical inertness of Ngs. After this finding, a vast series of Ng compounds were either characterized experimentally or predicted to be viable *in silico* (Thompson and Andrews, 1994; Pettersson et al., 1995, 1997, 1998a,b; Evans and Gerry, 2000a,b; Evans et al., 2000a,b; Khriachtchev et al., 2000, 2001, 2003a,b; Li et al., 2002; Feldman et al., 2003; Tanskanen et al., 2003; Cooke and Gerry, 2004a,b; Wang et al., 2004, 2012; Smith et al., 2007; Chakraborty et al., 2010; Pan et al., 2013a,b, 2014; Debackere et al., 2014; Khatua et al., 2014; Mondal and Chattaraj, 2014; Chakraborty and Chattaraj, 2015a,b; Pan et al., 2015a,b,c,d, 2016a,b, 2018a,b; Saha et al., 2015, 2016, 2017, 2019; Jana et al., 2018a). Similarly, the noble metals (M = Cu, Ag, Au) are known to be comparatively less reactive that allows their use in day-to-day life. Following the successful synthesis of different types of compounds containing Ng atoms, the possible union of noble metal and Ng atoms acquired appreciable attention. In Pyykkö (1995) showed the possibility of NgAu^+^ and NgAuNg^+^ through coupled-cluster based technique and in a subsequent study these species were characterized employing mass spectroscopic study (Schröder et al., 1998). Thereafter, a series of Ng complexes with Ng-Au bonds have been studied both experimentally and theoretically (Pan et al., 2015e, 2016c,d; Ghara et al., 2016; Jana et al., 2016, 2018b,c). The experimental and theoretical advancement in the complexes having Ng-Au bond has been summarized in a recent review paper (Pan et al., 2019).

In recent times, frustrated Lewis pairs (FLP) (Stephan, 2008) have seen an upsurge of interest since they can be used as metal-free catalysts, which can also activate some small molecules. The FLP skeleton is constructed by a pair of Lewis acid and base. These acidic and basic centers may be located in two separate molecules or in one particular molecule. Due to steric hindrance those centers are not allowed to form the usual dative bonds. Accordingly, further reactivity is exhibited by the unquenched Lewis acid and base centers. Welch et al. (2006) and Welch and Stephan (2007) reported the reversible activation of molecular hydrogen by an intramolecular boron/phosphorous FLP for the first time in 2006. After that several theoretical (Rokob et al., 2008, 2009; Hamza et al., 2009; Mueck-Lichtenfeld and Grimme, 2012; Ghara et al., 2019) studies were performed to understand the function of FLP in the cleavage of H-H bond in H_2_ molecule. Two different models of H_2_ activation by FLPs are proposed viz., electron transfer (ET) model as given by Rokob et al. (2008) and electric field (EF) model as given by Mueck-Lichtenfeld and Grimme (2012). Various imines, nitriles, enamens, alkenes, ketone, CO_2_, etc., have been catalytically hydrogenated by exploiting the hydrogen activating ability of FLPs (Chase et al., 2007, 2008; Spies et al., 2008; Sumerin et al., 2008; Ashley et al., 2009; Mahdi and Stephan, 2014, 2015; Stephan and Erker, 2015; Ghara and Chattaraj, 2019; Ghara et al., 2019). FLPs may also activate other molecules such as NO, SO_2_, CO_2_, N_2_O, CO, C_2_H_4_, C_2_H_2_ etc. (Mc Cahill et al., 2007; Moemming et al., 2009; Dureen and Stephan, 2010; Appelt et al., 2011; Cardenas et al., 2011; Kolychev et al., 2012; Sajid et al., 2014; Stephan and Erker, 2014; Ghara and Chattaraj, 2018a,b). FLP assisted small molecule activation thereby offers new synthetic opportunities. This performance of FLPs has rendered it as a highly attractive species being inexpensive and environment friendly. Recently, Erker et al. have shown that an intramolecular vicinal P/B FLP stabilized AuCl and AuNTf_2_ may surve as catalysts for the hydroamination of alkynes (Ueno et al., 2019). An active catalytic species [(FLP)Au]^+^ is generated in the reaction medium. As the Lewis acidic boron center interacts with the Au center it enhances the electrophilicity of the Au center, which in turn improves the catalytic activity of [(FLP)Au]^+^ species as suggested by Erker.

In the present study, the Ng (Ng = Ar-Rn) binding ability at the Au center of [(FLP)Au]^+^ is computationally investigated. Bond dissociation energy (*D*_0_), enthalpy (ΔH) and Gibbs free energy changes (ΔG) are calculated to evaluate how the [(FLP)Au]^+^ species can bind the Ng atoms. Topology of the electron density (Bader, 1990), natural bond orbital (NBO) (Reed and Weinhold, 1983), and energy decomposition (EDA) are analyzed and natural orbital for chemical valence (NOCV) (Morokuma, 1977; Mitoraj and Michalak, 2007; Mitoraj et al., 2009; Hopffgarten and Frenking, 2012) theory is to analyze the type of the bond formed connecting the Ng and the Au centers.

## Computational Details

Geometries of all the molecules have been optimized in the gas phase at the M06-2X-D3 (Zhao and Truhlar, 2008; Grimme et al., 2010)/def2-TZVP level and not applying any symmetry constraint. The functional M06-2X performs well in describing the FLP chemistry as reported previously (Huang et al., 2014). Effective core potential (Peterson et al., 2003) was used in order to take care of the relativistic effect in Au, Xe and Rn. It may be noted that the presence of the dispersion term is just adding a semi-empirical correction to the total Kohn-Sham energy and is important when the long-range interactions are strong (Grimme et al., 2010). It is expected to increase the contribution of the dispersion term and hence the overall interaction energy. Effectiveness of the MO6-2X-D3 in certain cases has been shown earlier albeit with the presence of some amount of medium range interaction in MO6-2X (Burns et al., 2011) Vibrational frequencies are calculated to confirm whether the stationary points belong to a minimum on the potential energy surface (PES) or a higher order saddle point. This techniques also provides the zero point energy (ZPE) and the thermodynamic corrections at 298.15 K temperature. Natural bond orbitals(NBO) were analyzed to obtain the partial natural charges on each atomic site and the Wiberg bond index (WBI) (Wiberg, 1968) value. All the calculations were done using Gaussian 16 suit of program (Frisch et al., 2016). Quantum theory of atoms in a molecule (QTAIM) is used to analyze the distribution of the electron density by using a Multiwfn software (Lu and Chen, 2012). An all electron basis set WTBS was made use of for Au, Xe and Rn for this computation.

The bonding situations were further analyzed by means of the EDA-NOCV method as provided in the (ADF, 2018).105 program package. The EDA-NOCV calculations have been carried out at the BP86-D3(BJ)/TZ2P-ZORA level with the M06-2X-D3/def2-TZVP optimized geometries. BP86 functional is selected since D3 or D3(BJ) is not compatible with M06-2X functional as used in ADF to get the dispersion contribution. In this analysis, the interaction energy (Δ*E*_int_) within two fragments may be decomposed into different energy components like:

(1) $$\begin{array}{l} \Delta {\text{E}}_{\text{int}}=\Delta {\text{E}}_{\text{elstat}}+\Delta {\text{E}}_{\text{Pauli}}+\Delta {\text{E}}_{\text{orb}}+\Delta {\text{E}}_{\text{disp}} \end{array}$$

Since we have used D3(BJ), it provides extra dispersion contribution between two interacting fragments. The term Δ*E*_orb_ comes from orbital mixing. It may be devided into individual components belonging to separate irreducible representations

(2) $$\begin{array}{l} \Delta {\text{E}}_{orb}=\underset{r}{\sum }\Delta {\text{E}}_{r} \end{array}$$

An analysis containing the EDA and the NOCV allows one to partition the net orbital interactions into the corresponding pairwise contributions. The deformation in charge density Δρ_*k*_(*r*), with orbital pairs Δρ_k_(r) and ψ_−k_(r) get mixed up provides the amount and the direction of the charge flow (Equation 3), and the related energy term Δ*E*_orb_ gives the amount of stabilization in orbital energy (Equation 4).

(3) $$\begin{array}{l} \Delta \rho_{orb}\left(r\right)=\sum \Delta \rho_{k}\left(r\right)=\overset{N/2}{\underset{k=1}{\sum }}\nu_{k}\left[-\varphi_{-k}^{2}\left(r\right)+\varphi_{k}^{2}\left(r\right)\right] \end{array}$$

(4) $$\begin{array}{l} \Delta {\text{E}}_{orb}=\underset{k}{\sum }\Delta {\text{E}}_{k}^{orb}=\overset{N/2}{\underset{k=1}{\sum }}v_{k}\left[-F_{-k,\;-k}^{TS}+F_{k,k}^{TS}\right] \end{array}$$

## Results and Discussion

**Figure 1:**
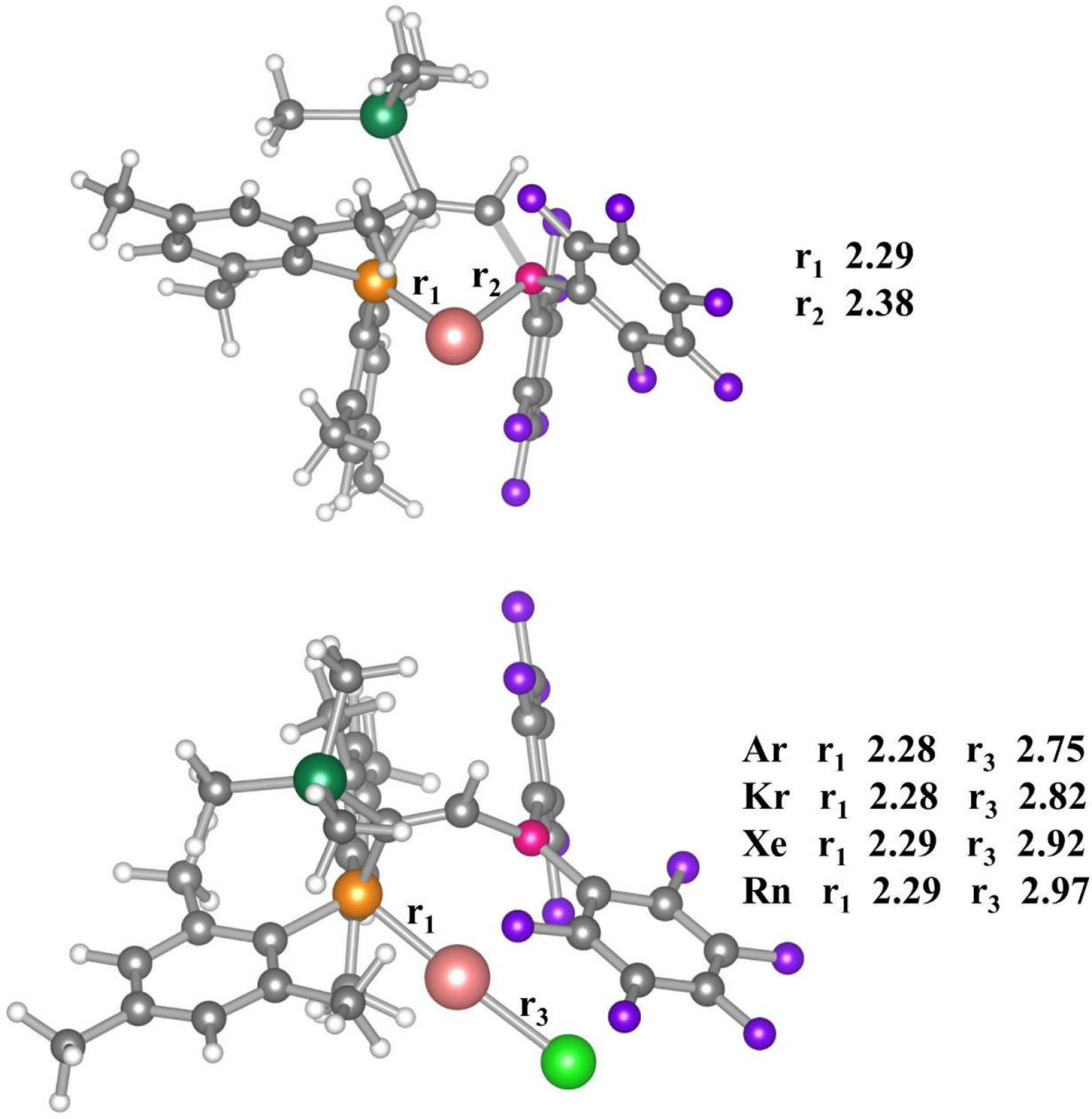
Optimized geometries of [(FLP)Au]^+^ (above) and [(FLP)AuNg]^+^ (below) complexes. Here, r_1_, r_2_, and r_3_ stands for Au-P, Au-B, and Au-Ng bond distances (in Å unit) calculated at M062X-D3/def2TZVP level of theory. Color code: Yellow for P, Pink for B, Merun for Au, Gray for C, White for H, Violet for F, Olive for Si and Green for Ngs atoms respectively.

The optimized geometries of the bare [(FLP)Au]^+^ complex and the Ng bound [(FLP)AuNg]^+^ complexes are displayed in Figure 1. The bare [(FLP)Au]^+^ complex corresponds to the *C*_1_ point group with ^1^A electronic state. The computed Au-P and Au-B bond distances are 2.29 and 2.38 Å respectively, whereas the experimental bond distances of these bonds are 2.27 and 2.35 Å respectively when a chloride ion is attached to the Au center of the complex (Ueno et al., 2019). The Au center posseses a natural charge of 0.42 |*e*|, and the P and B centers possess the NBO charge of 1.17 |*e*| and 0.60 |*e*|, respectively in this complex. Low positive charge on Au center is a consequence of P → Au donation and B → Au and P → Au backdonation (vide infra). The WBI values of Au-P and Au-B bonds are 0.69 and 0.32, respectively, implying partial covalent character of these bonds. The Au-P and Au-B bonds are further examined by electron density and EDA-NOCV analyses (*vide infra*).

Thus, any anionic/neutral ligand near the Au center may be polarized fascilitated by Au and can form chemical bond with Au. Accordingly, the [(FLP)Au]^+^ complex is enable to bind Ng atoms (Kr-Rn) effectively as discussed in this study. The [(FLP)AuNg]^+^ (Ng = Ar-Rn) complexes do not change the same electronic state and point group as that of the bare [(FLP)Au]^+^ complex. The Ng-Au bond dissociation energy (ZPE corrected, D_0_) values become 6.0–13.3 kcal/mol (see Table 1). Note that, the D_0_ values are gradually increasing down the group from Ar to Rn and it was expected as the polarizabilities of the Ng atoms are increasing down the group which help the Au center to deform the electron cloud of Ngs. Further, the thermochemical stabilities of the Ng bound [(FLP)Au]^+^ complexes are explored by calculating ΔH and ΔG associated with the dissociation of [(FLP)AuNg]^+^ into [(FLP)Au]^+^ and the respective Ng atoms at 298 K. The computed ΔH of all the dissociation processes is positive implying all these dissociation processes are endothermic and it also increases gradually down the group. The computed ΔG of dissociation is also positive for Kr-Rn bound cases implying endergonic dissociation of these Ng atoms from [(FLP)AuNg]^+^ (Ng = Kr-Rn) systems except the dissociation of Ar from [(FLP)AuAr]^+^ which is exergonic. Note that, all the dissociation processes are entropy driven and this is the reason for the observed exergonicity in the dissociation of [(FLP)AuAr]^+^ into [(FLP)Au]^+^ and the Ar atom. However, this dissociation could be frozen by slightly lowering the temperature. The HOMO-LUMO energy difference where HOMO and LUMO are the highest occupied and lowest unoccupied molecular orbitals respectively, is related to the global hardness (Parr and Chattaraj, 1991) can also be used to describe the stability of a molecular system. If the HOMO-LUMO gap (ΔE_H−L_) of a molecule is high, then it will not like to give or take electron. It means the system is relatively more stable. Since, the Δ*E*_H−L_ of [(FLP)Au]^+^ was 5.21 eV and after the Ng binding it increases to 5.45–5.59 eV which shows that the stability of the complex is increased upon Ng binding.

**Table 1:** ZPE corrected dissociation energy (D_0_ kcal/mol), enthalpy (ΔH, kcal/mol) and free energy (ΔG, kcal/mol) changes at 298 K for the dissociation process [(FLP)AuNg]^+^ → Ng + [(FLP)Au]^+^ (Ng = Ar-Rn), HOMO-LUMO energy differences (ΔE_H-L,_ eV), NPA charges at Au and Ng centers (q, au), WBI of Au-P (WBI_Au-P_) and Au-Ng (WBI_Au-Ng_) bonds calculated at M062X-D3/def2TZVP level.

**Complex**	**D_**0**_**	**ΔH**	**ΔG**	**Δ*E*_H-L_**	**q(Au)**	**q(Ng)**	**WBI_**Au-P**_**	**WBI_**Au-Ng**_**
[(FLP)Au]^+^				5.21	0.42		0.692	
[(FLP)AuAr]^+^	6.0	6.1	−1.6	5.58	0.25	0.11	0.752	0.187
[(FLP)AuKr]^+^	8.8	8.9	1.2	5.59	0.21	0.16	0.735	0.256
[(FLP)AuXe]^+^	11.9	12.0	4.2	5.58	0.17	0.22	0.710	0.348
[(FLP)AuRn]^+^	13.3	13.3	5.8	5.58	0.16	0.23	0.705	0.366

The NPA charges of the atoms in the complexes as provided by the NBO analysis are also listed in Table 1. In [(FLP)AuNg]^+^ complexes the Ng centers gain some positive NPA charges and at the same time the positive charge of the Au center decreases from 0.42 |*e*| to 0.25-0.16 |*e*| as a result of Ng binding. This shows that some charge transfer takes place from Ng to the Au center and also to the complex and the amount of charge transfer gets increased as the size of the Ng atoms. The WBI value of a chemical bond gives the idea about the degree of covalency of that bond. A smaller value of WBI of a chemical bond implies non-covalent type of interaction like electrostatic or van der Waals interaction. Conversely, a higher value of WBI of a chemical bond implies dominant covalent character of that bond. In our case, the WBI values of the Au-Ng bonds are gradually increasing from 0.187 (of Ar) to 0.366 (of Rn) implying a partial covalent character of the bonds. However, the WBI value of the Au-P bond increases slightly after Ng binding to the Au center, whereas it is not found between the Au and B centers of the FLP and these are evident from the Au-P and Au-B bond distances. Since, the Au-P bond distances remain almost same and the Au-B distance increase ~0.54–0.63 Å after the Ng binding.

The type of Au-P and Au-B bonds in the [(FLP)Au]^+^ complexes and the Au-P and Au-Ng bonds in the [(FLP)AuNg]^+^ complexes is understood through a topological electron density analysis and the results are given in Table 2. If the Laplacian of electron density [∇^2^ρ(r_c_)] is negative at the bond critical point (BCP) of a chemical bond it implies that the electron density is accumulated in between the two bonded atoms and thus a covalent bond is present between them. Conversely, a positive ∇^2^ρ(r_c_) value at the BCP of a bond implies non-covalent interaction between the two bonded atoms. Although, this hypothesis can provide an explanation of the nature of bonding in most of the cases, it cannot properly interpret the bond involving heavier transition metals (Macchi et al., 1998) It also fails to interprit the bond in some typical covalent molecules like CO and F_2_ (Cremer and Kraka, 1984). In that case, if the total electron energy density [H(r_c_)] is negative at the BCP of a chemical bond, then the bond may contain some short of shaired interaction as suggested by Cramer. Here, H(r_c_) is the sum of the local kinetic energy density [G(r_c_)] and the local potential energy density [V(r_c_)].

**Table 2:** Electron density descriptors at the bond critical points (BCP) of P-Au and B-Au bonds in [(FLP)Au]^+^ and of P-Au and Ng-Au bonds in [(FLP)AuNg]^+^ complexes (Ng = Ar-Rn) obtained at the M062X-D3/def2TZVP/WTBS level.

**Complex**	**BCP**	**ρ(r_**c**_)**	****∇^2^ρ**(r_**c**_)**	**G(r_**c**_)**	**V(r_**c**_)**	**H(r_**c**_)**	**–G(r_**c**_)/ V(r_**c**_)**
[(FLP)Au]^+^	P-Au	0.107	0.135	0.081	−0.129	−0.048	0.630
	B-Au	0.050	0.049	0.028	−0.045	−0.016	0.638
[(FLP)AuAr]^+^	P-Au	0.111	0.137	0.085	−0.136	−0.051	0.626
	Ar-Au	0.027	0.136	0.032	−0.030	0.002	1.067
[(FLP)AuKr]^+^	P-Au	0.110	0.140	0.085	−0.135	−0.050	0.629
	Kr-Au	0.032	0.128	0.033	−0.033	−0.001	0.983
[(FLP)AuXe]^+^	P-Au	0.109	0.141	0.084	−0.133	−0.049	0.633
	Xe-Au	0.031	0.117	0.030	−0.030	−0.001	0.981
[(FLP)AuRn]^+^	P-Au	0.109	0.142	0.084	−0.133	−0.049	0.634
	Rn-Au	0.032	0.111	0.029	−0.031	−0.001	0.954

In the bare [(FLP)Au]^+^ complex, although the ∇^2^ρ(r_c_) is positive at the BCPs of P-Au and B-Au bonds, the negative H(r_c_) values of the same indicate the partially shared bonding interactions. Furthermore, the value of –G(r_c_)/V(r_c_) is also a usefull quantity to describe the nature of a chemical bond. If the value of –G(r_c_)/V(r_c_) at the BCP of a chemical bond ranges in between 0.5 and 1 then it contains some degree of shared interaction (Ziolkowski et al., 2006). Conversely, for a purely non-covalent interaction the value of –G(r_c_)/V(r_c_) will be >1. Since, the value of –G(r_c_)/V(r_c_) lies within 0.5 and 1 for both the P-Au and B-Au bonds, some degree of shared interactions is present in these bonds. Now, in [(FLP)AuAr]^+^ complex, both the ∇^2^ρ(r_c_) and H(r_c_) are positive at the BCP of Au-Ar bond and so it may be classicified as non-covalent. Moreover, the value of –G(r_c_)/V(r_c_) is >1 at the BCP implying a purely non-covalent interaction between them. On the other hand, in [(FLP)AuNg]^+^ complexes (Ng = Kr-Rn) at the BCPs of Au-Kr, Au-Xe and Au-Rn bonds although ∇^2^ρ(r_c_) is positive, the negative H(r_c_) values indicate the partially shared bonding interactions. In addition, the value of –G(r_c_)/V(r_c_) lies in between 0.5 and 1 which also implies some degree of shared interactions in these bonds. Moreover, the values of ∇^2^ρ(r_c_), H(r_c_) and –G(r_c_)/V(r_c_) of the P-Au bond remain as in the bare complex after Ng binding exhibiting a partially covalent nature of the bond. Although, the ∇^2^ρ(r_c_), H(r_c_) and –G(r_c_)/V(r_c_) values of the B-Au bond in the bare complex indicate a partial covalent character of this bond, no BCP was found in between the Au and B centers in the [(FLP)AuNg]^+^ complexes.

**Figure 2:**
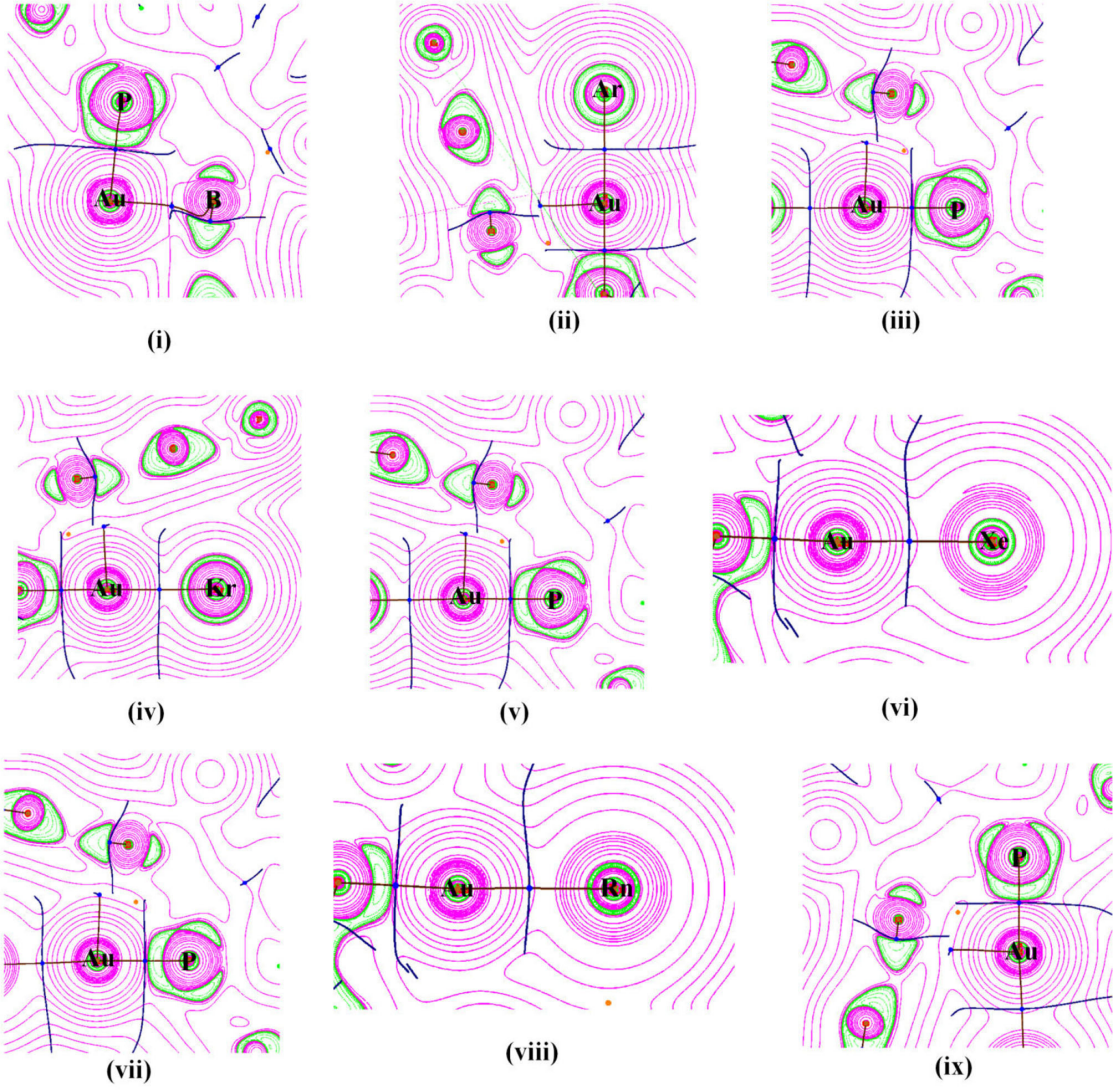
Contour plots of Laplacian of electron density [∇^2^ρ(r)] of (i) Au-P and Au-B bonds in [(FLP)Au]^+^, (ii) Au-Ar and (iii) Au-P bonds in [(FLP)AuAr]^+^, (iv) Au-Kr and (v) Au-P bonds in [(FLP)AuKr]^+^, (vi) Au-Xe and (vii) Au-P bonds in [(FLP)AuXe]^+^, (viii) Au-Rn and (ix) Au-P bonds in [(FLP)AuRn]^+^ at the M06-2X-D3/def2-TZVP/WTBS level. [Pink solid lines stand for ∇^2^ρ(r) > 0 and the green dotted lines stand for ∇^2^ρ(r) < 0].

The contour plots of ∇^2^ρ(r) of P-Au and B-Au bonds in the bare complex [(FLP)Au]^+^ and P-Au and Au-Ng bonds in the Ng bound complexes are provided in Figure 2. In these figures solid pink lines depict the portion with positive ∇^2^ρ(r), the dotted green lines depict the region with negative ∇^2^ρ(r) and the blue solid lines represent the interbasin paths between the two atomic basins. It is apparent from all these plots that there is no green region which belongs to the interbasin path. Only, the valence orbitals are slightly deformed in shape.

The nature of the Au-P and Au-B bonds present in the [(FLP)Au]^+^ complex is further analyzed by EDA and the results are depicted in Table S1 in the Supportion information (SI). The contributions of Δ*E*_elstat_ and Δ*E*_orb_ toward the total attractive interaction between the FLP and the Au^+^ cation become 53.1 and 41.5% respectively, whereas the Δ*E*_disp_ has a very less contribution (5.4%). Hence, the complex is mostly stabilized by electrostatic interaction followed by the orbital interactions between the FLP and the Au^+^ cation. Decomposition of Δ*E*_orb_ into pair-wise orbital interactions shows that there exist two major contributing orbital interactions Δ*E*_orb(1)_ and Δ*E*_orb(2)_, and four minor contributing orbital interactions Δ*E*_orb(3)_ to Δ*E*_orb(6)_. The deformation densities associated with these orbital interaction channels are also depicted in Figure S1 in the SI to understand the origin of these orbital interactions. The deformation density Δρ(1) (associated with Δ*E*_orb(1)_) refers to the amount of electron density donated from the Lewis basic P center to the Au^+^ cation, whereas Δρ(2) (associated with Δ*E*_orb(2)_) refers to the amount of electron density donated backward from the Au^+^ cation to both the LA and LB centers of the FLP. The remaining four deformation densities Δρ(3) to Δρ(6) also refer to the backward donation of electron density from the Au^+^ cation to the FLP and these are very small.

**Figure 3:**
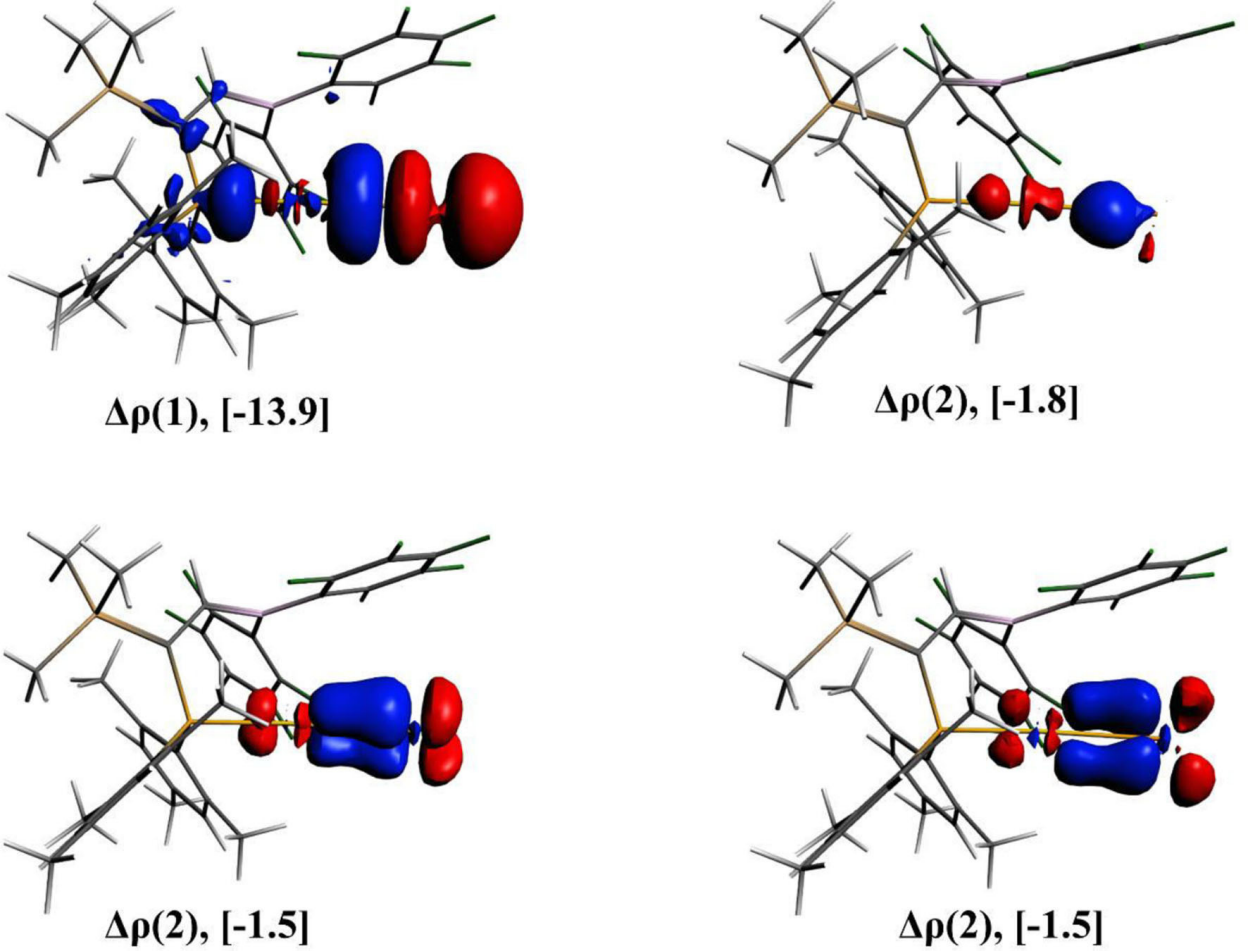
The plots of deformation densities (Δρ) of the pair-wise orbital interactions and the associated Δ*E*_orb_ energies obtained from the EDA-NOCV calculation on [(FLP)AuXe]^+^. The color code of charge flow is red → blue.

The nature of Au-Ng bonds present in the [(FLP)AuNg]^+^ complexes is also analyzed by EDA and the results are given in Table 3. In these Au-Ng bonds, the contribution of the Δ*E*_elstat_ and Δ*E*_orb_ are almost equal (~40%) to the total attractive interaction between the Ng and the [(FLP)Au]^+^ complex, and the contribution from the Δ*E*_disp_ is less but not negligible (18–19%). However, the total attractive interaction made by these three interactions is sufficient to overcome the repulsive Pauli interaction and therefore the Δ*E*_int_ becomes negative. Further, decomposing Δ*E*_orb_ into pair-wise orbital interactions by NOCV method we get four different orbital interaction channels Δ*E*_orb(1)_ to Δ*E*_orb(4)._ Among them, the Δ*E*_orb(1)_ has the maximum contribution (68–73%) to the total Δ*E*_orb_ of all the Au-Ng bonds. The deformation densities viz., Δρ(1), Δρ(2), Δρ(3), and Δρ(4) associated with the Δ*E*_orb(n)_ (*n* = 1–4) of the [(FLP)AuXe]^+^ complex are also depicted in Figure 3. Here, the Δρ(1) (corresponding to the major contributing Δ*E*_orb(1)_) refers to the donation of σ-electron density from the Xe atom to the Au center. Conversly, the Δρ(2) refers to the back donation of σ-electron density from the Au center to the Xe atom. Moreover, the Δρ(3) and Δρ(4) refer to the back donation of π-electron density from the Ng to the Au center. However, the donation of σ-electron density from the Xe atom to the Au center is not fully compensated by the sum of the back donations of electron density from the Au center to the Xe atom which leads to the observed positive NPA charge on the Xe center in the [(FLP)AuXe]^+^ complex.

**Table 3:** Energy decomposition analysis (EDA) results for the [(FLP)AuNg]^+^ complexes by taking Ng as one fragment and [(FLP)Au]^+^ as another, studied at the BP86-D3(BJ)/TZ2P//M06-2X-D3/ def2TZVP level. All energy values are given in kcal/mol.

**Energy**	**[(FLP)AuAr]^**+**^**	**[(FLP)AuKr]^**+**^**	**[(FLP)AuXe]^**+**^**	**[(FLP)AuRn]^**+**^**
Δ*E*_int_	−6.0	−11.3	−16.7	−19.4
Δ*E*_Pauli_	18.9	24.8	32.5	36.1
Δ*E*_disp_^[a]^	−4.9 (19.8%)	−6.6(18.2%)	−8.9 (18.0%)	−10.1 (18.2%)
Δ*E*_elstat_^[a]^	−10.0 (40.3%)	−14.4 (40.0%)	−20.3 (41.3%)	−23.3 (41.9%)
Δ*E*_orb_^[a]^	−9.9 (39.8%)	−15.1 (41.7%)	−20.1 (40.7%)	−22.2 (39.9%)
Δ*E*_orb(1)_^[b]^	−6.8 (68.7%)	−9.7 (64.2%)	−13.9 (69.3%)	−16.2 (73.0%)
Δ*E*_orb(2)_^[b]^	−1.0 (10.4%)	−1.4 (9.3%)	−1.8 (9.1%)	−1.8 (7.9%)
Δ*E*_orb(3)_^[b]^	−0.8 (8.2%)	−1.2 (7.6%)	−1.5 (7.6%)	−1.6 (7.0%)
Δ*E*_orb(4)_^[b]^	−0.8 (7.9%)	−1.1 (7.5%)	−1.5 (7.7%)	−1.6 (7.1%)

[a] *The values inside parentheses refer to the percentage contributions toward the total attractive interactions ΔE_elstat_ + ΔE_orb_ + ΔE_disp_*.

[b] *The values inside parentheses correspond to the percentage contributions toward the total orbital interactions, ΔE_orb_*.

## Conclusion

The noble gas (Ng) binding ability of a monocationic [(FLP)Au]^+^ species has been assessed. Here, the monocationic [(FLP)Au]^+^ species is formed by coordinating Au(I) cation with the LA and LB centers of a FLP. The Au-P and Au-B bonds in [(FLP)Au]^+^ are partially covalent in nature as revealed by electron density and energy decomposition analyses. The Au center possesses an NPA charge of 0.42 |*e*| as obtained from NBO analysis.

The bond dissociation energy values (ZPE corrected, D_0_) for the dissociation of Au-Ng bonds (Ng = Ar-Rn) get increased and range between 6.0 and 13.3 kcal/mol from Ar to Rn. It is apparent that the dissociation of Au-Ng bonds is endothermic as well as endergonic for Ng = Kr-Rn, whereas the same for Ng = Ar is endothermic but exergonic at room temperature.

The stability of the complex is increased upon Ng binding as highlighted through an increase in the HOMO-LUMO gap (ΔE_H−L_) of [(FLP)Au]^+^, as a result of Ng binding. The partial covalent character of the Au-Ng bonds is indicated by the WBI values as well as through an electron density analysis at the BCP of these bonds. The Ng atoms get a slightly positive charge of 0.11–0.23 |*e*|, which indicates some amount of charge transfer from it. EDA demonstrates that both electrostatic and orbital interactions contribute almost equally to the total interaction energy between the Ng and the [(FLP)Au]^+^ complex, and the dispersion interaction has a very less contribution. Further, decomposing the orbital interaction (Δ*E*_orb_), we obtained four different interaction channels (Δ*E*_orb(n)_, *n* = 1–4), which are the results of donation and back donation of electron densities between Ng and Au centers as obtained from the EDA-NOCV calculation.

## Data Availability Statement

The raw data supporting the conclusions of this article will be made available by the authors, without undue reservation.

## Author Contributions

MG has done the computation and has written the first draft of the manuscript. PC has critically examined the project and has made the final corrections. Both authors contributed to the article and approved the submitted version.

## Conflict of Interest

The authors declare that the research was conducted in the absence of any commercial or financial relationships that could be construed as a potential conflict of interest.
