# Supplementary material for: Noble Gas Binding Ability of an Au(I) Cation Stabilized by a Frustrated Lewis Pair: A DFT Study
Source: Front Chem. 2020 Jul 21;8:616. doi: 10.3389/fchem.2020.00616 (PMC7396548; doi:10.3389/fchem.2020.00616)
Supplement: Supplementary file 1 [file Table_1.DOCX]

**Supporting Information**

**Table S1.** Energy decomposition analysis (EDA) results for the [(FLP)Au]^+^ complexes by taking Au^+^ as one fragment and FLP as another, studied at the BP86-D3(BJ)/TZ2P+//M06-2X-D3/ def2TZVP level. All energy values are given in kcal/mol.

| **Energy** | [(FLP)Au]^+^ |
| --- | --- |
| ∆*E*_int_ | -161.7 |
| ∆*E*_Pauli_ | 227.8 |
| ∆*E*_disp_^[a]^ | -21.1 (5.4%) |
| ∆*E*_elstat_^[a]^ | -206.9 (53.1%) |
| ∆*E*_orb_^[a]^ | -161.5 (41.5%) |
| ∆*E*_orb(1)_^[b]^ | -82.8 (51.3%) |
| ∆*E*_orb(2)_^[b]^ | -23.1 (14.3%) |
| ∆*E*_orb(3)_^[b]^ | -9.3(5.7%) |
| ∆*E*_orb(4)_^[b]^ | -8.5(5.3%) |
| ∆*E*_orb(5)_^[b]^ | -7.5(4.6%) |
| ∆*E*_orb(6)_^[b]^ | -5.8(3.6%) |

^a^The values in parentheses are the percentage contributions to the total attractive interactions ∆*E*_elstat_ + ∆*E*_orb_ + ∆*E*_disp._ ^b^The values in parentheses are the percentage contributions to the total orbital interactions ∆*E*_orb_.


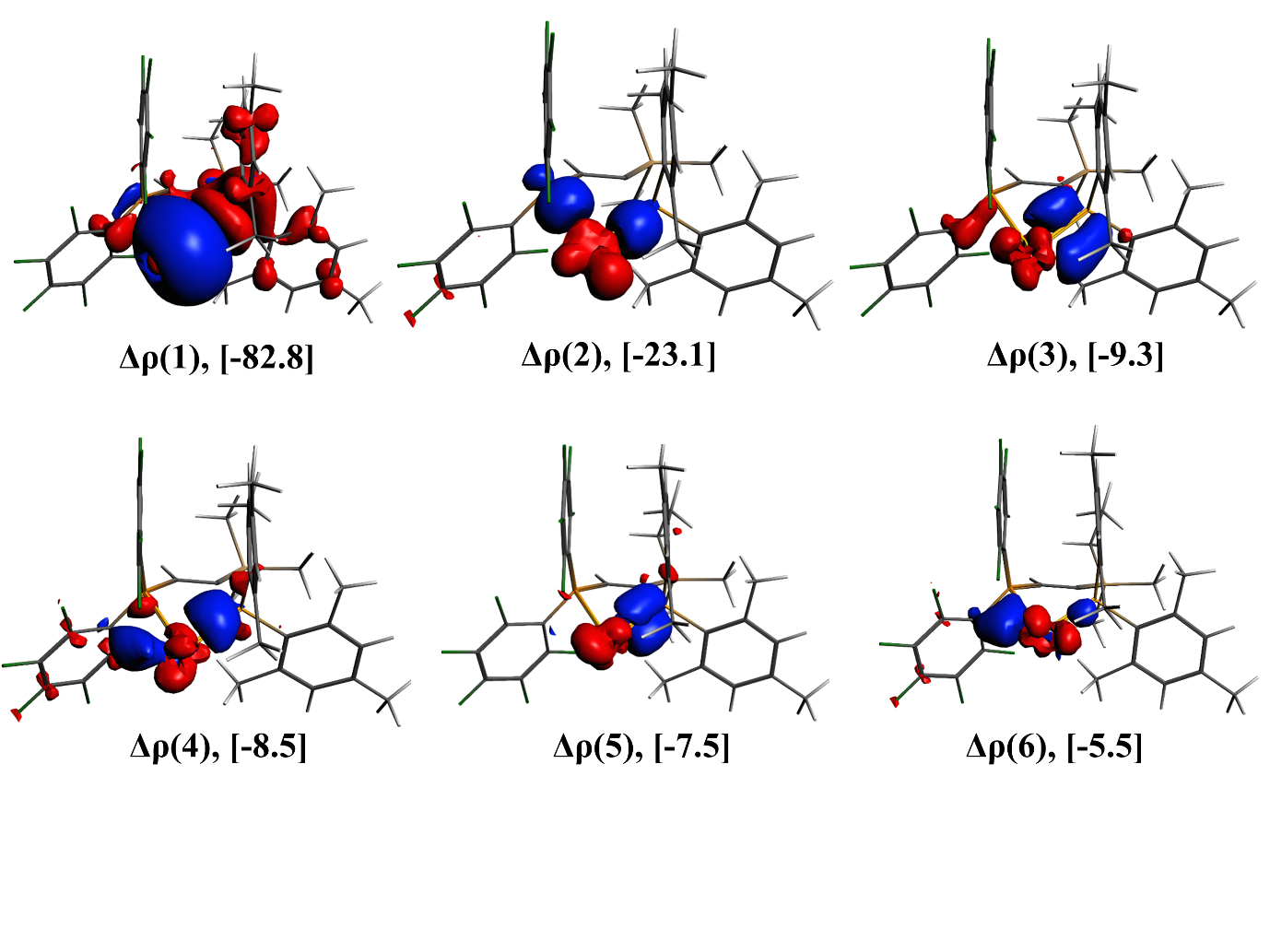


**Figure S1.** The plots of deformation densities (Δρ) of the pair-wise orbital interactions and the associated ∆*E*_orb_ energies obtained from the EDA-NOCV calculation on [(FLP)Au]^+^. The color code of charge flow is red → blue.
